# Long Noncoding RNA Regulation of Pluripotency

**DOI:** 10.1155/2016/1797692

**Published:** 2015-11-30

**Authors:** Alessandro Rosa, Monica Ballarino

**Affiliations:** ^1^Department of Biology and Biotechnology Charles Darwin, Sapienza University of Rome, 00185 Rome, Italy; ^2^Institute Pasteur Fondazione Cenci-Bolognetti, Sapienza University of Rome, 00185 Rome, Italy

## Abstract

Pluripotent stem cells (PSCs) represent a unique kind of stem cell, as they are able to indefinitely self-renew and hold the potential to differentiate into any derivative of the three germ layers. As such, human Embryonic Stem Cells (hESCs) and human induced Pluripotent Stem Cells (hiPSCs) provide a unique opportunity for studying the earliest steps of human embryogenesis and, at the same time, are of great therapeutic interest. The molecular mechanisms underlying pluripotency represent a major field of research. Recent evidence suggests that a complex network of transcription factors, chromatin regulators, and noncoding RNAs exist in pluripotent cells to regulate the balance between self-renewal and multilineage differentiation. Regulatory noncoding RNAs come in two flavors: short and long. The first class includes microRNAs (miRNAs), which are involved in the posttranscriptional regulation of cell cycle and differentiation in PSCs. Instead, long noncoding RNAs (lncRNAs) represent a heterogeneous group of long transcripts that regulate gene expression at transcriptional and posttranscriptional levels. In this review, we focus on the role played by lncRNAs in the maintenance of pluripotency, emphasizing the interplay between lncRNAs and other pivotal regulators in PSCs.

## 1. Introduction

The term long noncoding RNAs (lncRNAs) refers to a heterogeneous class of RNA polymerase II (Pol II) transcripts greater than 200 nucleotides in length with no evident protein-coding capacity [1, 2]. They are generally spliced from multiexonic precursors, capped, polyadenylated, and localized to the nucleus, cytoplasm, or both [2, 3]. Based on the anatomical properties of their transcription loci and the relationship with the adjacent genes, lncRNAs can be classified in intronic, intergenic, or overlapping (in sense or antisense orientation) transcripts. Even though lncRNAs are less conserved than mRNA and small noncoding RNAs [4, 5], lack of conservation does not imply a lack of function [6]. Indeed, both the transcript length and the versatility of RNA to base-pair let these molecules fold into complex secondary structures [7, 8], which are interspersed with longer and less conserved stretches of nucleotide sequences. As highlighted by pioneering studies [1, 6], these structures allow lncRNAs to simultaneously interact with multiple complexes, thereby coordinating their activities.

Even though only a part of lncRNA transcripts have been mechanistically characterized, several studies have shown the participation of lncRNAs in different processes related to normal physiology and/or disease [4, 6]. As they are Pol II transcripts, lncRNA expression can be tightly regulated. Indeed lncRNA transcripts are globally more tissue specific than protein-coding genes suggesting potential roles in specifying cell identity [9, 10].

The intracellular localization of lncRNAs is predictive of their mode of action [6, 10]. Usually, nuclear lncRNAs can guide chromatin modification complexes to specific genomic loci and/or serve as molecular scaffolds that tether together distinct functionally related complexes [11, 12]. Due to their intrinsic ability to base-pair with other nucleic acids, both* cis*-acting (on neighboring genes) and* trans*-acting (on distant loci) lncRNAs can exert either repressive or promoting activities on target genes by coordinating protein and RNA interactions [12–14]. Based on known examples [12], nuclear lncRNAs can exert their regulatory function as decoys by simply titrating transcription factors and other proteins away from chromatin [15–17]. As a paradigm, depletion of the lncRNA PANDA substantially increased target genes occupancy by NF-YA, a nuclear transcription factor that triggers apoptosis upon DNA damage [17]. lncRNA binding on DNA can initiate the formation of heterochromatin by recruitment of DNA or histone methyltransferases (such as the histone H3 lysine 27, H3K27, and methyltransferase complex PRC2), resulting in repression of gene expression. Conversely, transcriptional activation can be induced by recruitment of different chromatin modifiers, such as the H3 lysine 4, H3K4, and methyltransferase MLL1, or by changing the 3D chromatin conformation [12, 13]. Among the* cis*-acting species, the enhancer-associated ncRNAs (eRNAs) are functional transcripts participating in many programs of gene activation. In particular, they play fundamental roles in targeting chromatin-remodeling complexes to specific promoters and to assist the formation of chromatin loops [18]. Using an integrated epigenomic screening, Ounzain and colleagues recently established a catalogue of enhancer-associated noncoding RNAs dynamically expressed in ESCs during cardiac differentiation [19]. The expression of these transcripts correlated with the expression of target genes in their genomic proximity. Interestingly, the expression of the eRNAs was inhibited when the target mRNAs reached maximal levels. Overall, these data gave an important contribution to the functional impact of cardiac eRNAs on heart development and cardiac remodeling after injury.

Some other lncRNAs are localized in the cytoplasm, where they can regulate gene expression through base-pairing complementary regions on target RNAs. In human, several cytoplasmic lncRNAs transactivate Staufen1-mediated mRNA decay by duplexing with 3′UTRs via Alu elements [20]. Another example is represented by the *β*-site APP-cleaving enzyme 1 BACE1-AS antisense RNA, which binds to BACE1 mRNA inducing its stabilization. By regulating BACE1 expression, the noncoding RNA plays a role in controlling the boundaries between physiology and pathology driving Alzheimer's disease pathology [21]. Base-pairing is also the principle that applies to the competing endogenous RNA (ceRNA) activity of lncRNAs [22]. In this case, lncRNAs can indirectly enhance protein translation by sequestering, or “sponging,” miRNAs that otherwise would inhibit their target mRNAs. This mechanism has been shown to be involved in differentiation and cancer [22, 23]. Finally, a peculiar class of sponging lncRNAs is represented by circular RNAs (circRNAs) [24, 25], whose unusual circular structure confers increased stability. Altogether, these different properties engender lncRNAs to operate through distinct modes of action and to exert a wide range of functions across diverse biological processes.

Embryonic Stem Cells (ESCs) are the* in vitro* counterpart of the pluripotent epiblast of the blastocyst and constitute a useful system to study the molecular mechanisms at the basis of pluripotency. A group of transcription factors (TFs), comprising OCT4, NANOG, and SOX2, has been proposed as the core regulatory circuitry in ESCs [26]. These are pluripotency factors that ensure the proper expression of genes involved in the maintenance of the undifferentiated state. At the same time, they repress many genes that play a role during subsequent development. Such developmental genes, however, are often kept in a silent but “poised” state by the establishment of bivalent chromatin domains, where histone repressive marks coexist with marks related to active transcription [27]. It is now becoming clear that the core pluripotency TFs operate in concert with miRNAs and lncRNAs [28–30]. One example of a miRNA family that plays a role in the crossroad between pluripotency and differentiation is the miR-302 family [31]. Among other activities, miR-302 regulates the balance between agonists and antagonists of the TGF*β*/BMP signalling, which is a crucial pathway for the choice between maintenance of pluripotency and differentiation [32]. In ESCs, the activity of miR-302 is counteracted by let-7, an opposing miRNA family that plays a prodifferentiative role [33]. Other miRNAs also facilitate differentiation by targeting pluripotency factors or chromatin modifiers [28]. In this review, we focus on recent evidence suggesting that lncRNAs also play an important role in the maintenance of pluripotency.

ESCs have represented for a long time the only system to model human early development. More recently, the Nobel Prize-awarded derivation of induced Pluripotent Stem Cells (iPSCs) provided an alternative source of pluripotent cells [34]. iPSCs can be derived from human somatic adult cells through a reprogramming process consisting in the ectopic expression of defined factors. As their derivation requires a simple skin biopsy (or blood sampling), human iPSCs overcome ethical and legislative issues that limit the research based on human ESCs (hESCs). Importantly, iPSCs generated from human patients with genetic disorders represent a promising tool for both regenerative medicine and* in vitro* disease modeling.

## 2. The lncRNA Signature in Embryonic Stem Cells

As for protein-coding genes and miRNAs [31], Pluripotent Stem Cells express a characteristic set of lncRNAs. The lncRNA signature of mouse ESCs (mESCs) has been defined by microarray analysis [35] and genome-wide mapping of chromatin marks of actively transcribed genes, such as trimethylation of lysine 4 of histone H3 (H3K4me3) in the promoter coupled with trimethylation of lysine 36 of histone H3 in the transcribed region (K4-K36 domain) [36]. Work by Dinger et al. [35] identified several lncRNAs that are differentially expressed in proliferating mESCs and upon induction of hematopoietic differentiation. Analysis of K4-K36 domains located outside the known protein-coding loci allowed Guttman et al. [36] to identify over a thousand novel lncRNAs in mESCs and somatic cells. The catalogue of mESC lncRNAs was then expanded by including a substantial fraction of species transcribed from genes not marked by a K4-K36 domain, identified by a computational method that allowed the reconstruction of the whole transcriptome from RNA-Seq data (Scripture) [37]. A significant subset of these lncRNAs may be regulated at the transcriptional level by the ESC core TFs [29, 38].

As in the case of mouse ESCs, K4-K36 domains analysis allowed the initial identification of a characteristic set of lncRNAs genes expressed in human ESCs [39]. This list was then further extended by integrating data from RNA-Seq analysis [4]. A more detailed characterization has shown that some human lncRNAs could be under the direct control of the core pluripotency TFs [40, 41].

## 3. lncRNAs Play a Role in the Maintenance of Pluripotency in ESCs

Increasing evidence points to a crucial role for lncRNAs in the maintenance of ESC self-renewal (pluripotency), thus preventing their differentiation. In a large-scale functional study, the individual knockdown of more than 90% of lncRNAs (out of 147 tested) caused a significant perturbation of the transcriptome, often resulting in the loss of mESC pluripotency [29]. Interestingly, lncRNAs involved in the maintenance of ESC self-renewal are often transcriptionally regulated by core pluripotency TFs and act in regulatory networks. Examples of this mechanism include AKO28326/GOMAFU/MIAT (OCT4-activated) and AK141205 (NANOG-repressed) lncRNAs that when altered lead to robust changes in OCT4 and NANOG levels and affected pluripotency of mESCs [38]. The lncRNA TUNA/MEGAMIND is required for mESCs proliferation and maintenance of self-renewal [5]. TUNA binds a complex comprising several RNA-binding proteins and activates transcription of NANOG and SOX2 upon binding on their promoters [42]. The interplay between core TFs and lncRNAs has been reported also in hESCs for lncRNA_ES1, lncRNA_ES2, and lncRNA_ES3 [40]. Taken together, these examples indicate that lncRNAs are involved in the maintenance of the undifferentiated state and the repression of genetic programs that direct lineage commitment during differentiation.

**Figure 1:**
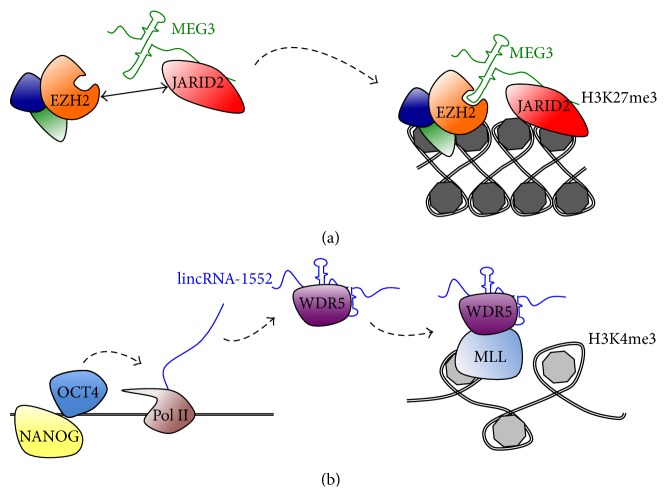
Examples of nuclear lncRNAs interacting with chromatin regulators in ESCs. (a) The lncRNA MEG3 promotes the interaction between the EZH2 subunit of PRC2 and JARID2, thus guiding PRC2 recruitment and H3K27me3 deposition at JARID2 target sites [47]. It has also been proposed that lncRNAs specify the target site recognition of PRC2/JARID2 via RNA-DNA base-pairing (not shown) [47]. (b) lincRNA-1552 transcription is promoted by core pluripotency factors and is required for ESC self-renewal [29]. This and other transcripts positively regulate TrxG activity by binding and stabilizing the WDR5 cofactor [52].

The challenge now is to dissect the molecular mechanisms underlying the functions of these ESC lncRNAs. Mechanistically, nuclear lncRNAs may exert their function by binding and regulating the activity and/or target specificity of chromatin-modifying factors. It has been shown that ESC lncRNAs interact with all classes of histone modifiers (writers, readers, and erasers), as well as other chromatin-associated proteins [29]. This is in line with a possible role of these long transcripts as molecular scaffolds that bridge together different chromatin modification complexes [11]. Recent examples support the hypothesis that lncRNAs may be pivotal regulators of the activity of crucial chromatin modifiers, which play an essential role in the epigenetic regulation of ESCs pluripotency and differentiation. Genome-wide analysis identified a multitude of potential lncRNA interactors of PRC2 in mESCs and a somewhat promiscuous RNA-binding activity of this complex has been suggested [43, 44]. Recent work proposed that lncRNA binding might be important to modulate the interaction of PRC2 with its cofactors, thus modulating its activity and/or specificity. One of such cofactors is JARID2, belonging to the JUMONJI family of lysine demethylases (KDMs). JARID2 is peculiar as its KDM catalytic domain is inactive and it is particularly enriched in ESCs where it regulates PRC2 activity and genome occupancy [45, 46]. It has been recently shown that JARID2 contains RNA-binding region and directly interacts with about 100 previously annotated lncRNAs in mESCs [47]. Particularly interesting, among these interactors are MEG3 (also known as GTL2), RIAN, and MIRG, lncRNAs that are encoded within an imprinted locus on chromosome 12qF1, referred to as the Dlk1-Dio3 gene cluster. Proper expression of these lncRNAs is required for embryonic development [48, 49] and to achieve full pluripotency during reprogramming, as iPSCs carrying aberrantly silenced Dlk1-Dio3 cluster genes are unable to fulfill stringent pluripotency tests, such as contribution to chimaeric mice development and complementation of a tetraploid blastocyst [50]. Functionally, by binding JARID2, MEG3 and other Dlk1-Dio3 gene cluster lncRNAs may modulate the activity of PRC2 in Pluripotent Stem Cells. Genome-wide analysis indeed showed that Meg3 stimulates PRC2 occupancy* in trans* at genomic loci encoding for factors involved in differentiation and development [44]. These genes are derepressed in human iPSC lines expressing low levels of MEG3, suggesting evolutionary conservation of the MEG3-JARID2 axis. Mechanistically, MEG3 and other lncRNAs work as scaffolds to increase the interaction between JARID2 and the PRC2 core component EZH2 and, therefore, PRC2 assembly on chromatin at JARID2 target sites. Moreover, it has been suggested that these lncRNAs may also guide the initial recruitment of PRC2/JARID2 at specific target sites in pluripotent cells via RNA-DNA base-pairing [47] (Figure 1).

Trithorax group (TrxG) factors, including mammalian MLL complexes, positively regulate transcription via the H3K4me3. This activity is required to maintain pluripotency in ESCs. In particular, the WDR5 member of the MLL complex directly interacts with the core transcriptional regulatory circuitry and its depletion causes loss of self-renewal [51]. By taking advantage of an RNA-binding deficient mutant, Yang and coworkers recently demonstrated that the interaction with RNA is essential for WDR5 activity [52]. The half-life of the WDR5 mutant protein in the nucleus is reduced compared to wild-type, indicating that RNA binding positively regulates protein stability. Over 1000 RNAs might bind WDR5 in ESCs, including 23 previously annotated lncRNAs. Among these interactors, six were previously identified as lncRNAs required to maintain pluripotency in mESCs [29], providing a mechanistic explanation of their function. WDR5 also binds the promoters of two of these interacting lncRNAs, lincRNA-1592 and lincRNA-1552, suggesting a* cis* regulatory mechanism [49]. lincRNA-1552 expression may be under the direct control of many pluripotency transcription factors, including OCT4, NANOG, and KLF4 that bind its promoter, and its knockdown leads to misexpression of OCT4 and NANOG, among other mRNAs [29]. This evidence, together with the impairment of self-renewal in cells expressing the RNA-binding deficient WDR5 mutant [52], suggests that lncRNAs interacting with the Trithorax complex play a crucial role in the maintenance of ESC pluripotency (Figure 1).

The interplay between lncRNAs and Trithorax complexes may also direct specification towards specific cell fates upon ESCs differentiation. The homeotic genes* Hoxa6* and* Hoxa7* are involved in the specification of mesoderm derived tissues and organs [53, 54]. Bertani and colleagues demonstrated that the lncRNA MISTRAL (MIRA) mediates the transcriptional activation of* Hoxa6* and* Hoxa7* genes by recruiting MLL to chromatin [55]. MIRA-mediated activation of* Hoxa6* and* Hoxa7* culminates in the expression of genes involved in early germ layer specification in differentiating mESCs. Another interesting example of lncRNA involved in mESC differentiation is pRNA, which is localized in the nucleolus [56]. In Pluripotent Stem Cells, chromatin is globally in a transcriptionally permissive open state and becomes increasingly condensed and transcriptionally repressed upon differentiation (reviewed in [57]). Chromatin condensation occurs also at ribosomal genes and is promoted by pRNA, which guides the nucleolar repressor factor TIP5/BAZ2A to ribosomal DNA (rDNA) [56]. Interestingly, pRNA overexpression caused an increase of heterochromatin also outside rDNA, initiating the global epigenetic remodeling normally observed during differentiation.

**Figure 2:**
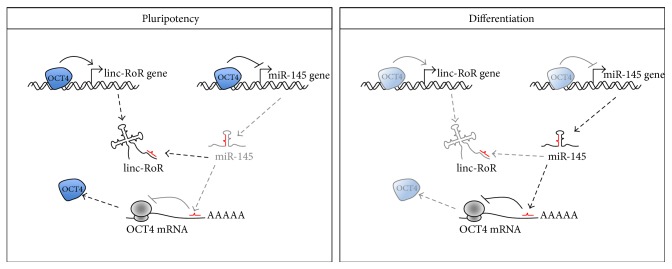
linc-RoR as a paradigm for lncRNA regulation of pluripotency in the cytoplasm. In pluripotent cells, the levels of linc-RoR are controlled by OCT4. In a positive feedback loop, linc-RoR sponges miR-145, thus derepressing its targets, including OCT4. OCT4 also negatively controls miR-145 at the transcriptional level [41, 59–61]. See text for details.

In addition to nuclear ESC lncRNAs, on the other side of the coin, fewer examples exist for cytoplasmic lncRNAs that regulate pluripotency. During the initial steps of the reprograming process, cells initiating their conversion to pluripotency must elude inhibitory hurdles, such as cell cycle arrest, senescence, and apoptosis, raised by p53 activation by the overexpression of the reprogramming factors [58]. Thus, any change in p53 activity is predicted to affect the efficiency of reprogramming by limiting the number of cells entering the process. In this context, the cytoplasmic linc-RoR (Regulator of Reprogramming) was initially identified as lncRNA able to promote the reprogramming process [41] by acting as a negative regulator of p53 [59]. Subsequently, Wang and colleagues showed that endogenous linc-RoR also plays a key role in the maintenance of hESC self-renewal by acting as a ceRNA [60]. Previous work had shown that a single miRNA, miR-145, inhibits translation of core TFs during ESC differentiation [61]. According to the model by Wang et al., in human ESCs linc-RoR would trap miR-145, derepressing the translation of the core pluripotency transcription factors OCT4, SOX2, and NANOG and ensuring proper levels of expression in undifferentiated hESC. Upon differentiation, the disappearance of linc-RoR releases miR-145, allowing it to repress the translation of core pluripotency factors [41]. Thus, this work strongly supports the idea that linc-RoR acts as a miRNA sponge. Since OCT4, at the transcriptional level, represses miR-145 and activates linc-RoR, these studies unraveled an interesting network comprising TFs, long and short regulatory RNAs which act at the crossroad between self-renewal and differentiation (Figure 2).

**Figure 3:**
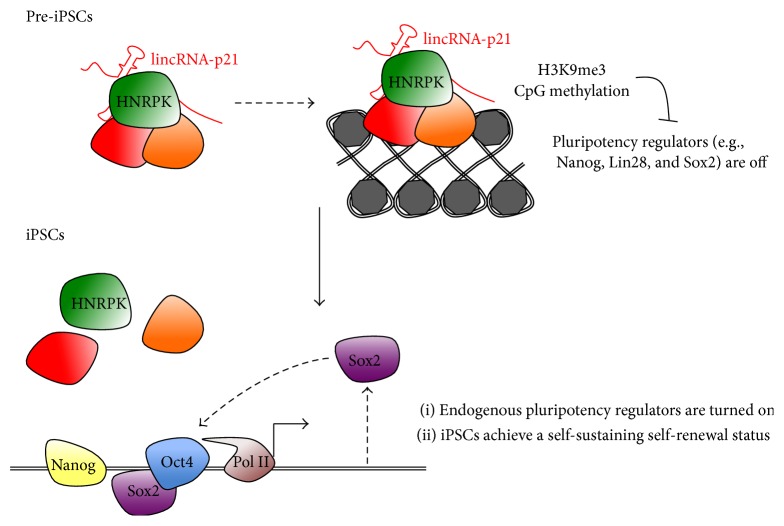
lincRNA-p21 regulates the transition of pre-iPSCs to iPSCs. During reprogramming, the induction of endogenous pluripotency genes is necessary to achieve a self-sustaining status in which the core regulatory factors act in a positive feedback loop on their own expression. lincRNA-p21 hinders this transition by recruiting an inhibitory complex, which deposits repressive marks such as H3K9me3 and CpG methylation on the promoters of pluripotency regulators [62].

More recently, Bao and colleagues [62] showed that lincRNA-p21, a nuclear noncoding transcript previously characterized as a global repressor of the p53-dependent transcriptional cascade [63], represents another example of lncRNA regulating pluripotency. Interestingly, in the context of somatic cell reprogramming, lincRNA-p21 inhibits this process without inducing apoptosis or impairing cell proliferation. It was identified in a functional screening performed in mouse to examine events accompanying the pre-iPSCs to iPSCs conversion. This is a late step, required to achieve a self-sustaining fully reprogrammed status, in which the cells become independent of the activity of the exogenous reprogramming factors and turn on the expression of endogenous pluripotency regulators [64]. Three lncRNAs, including lincRNA-p21, had a negative effect in pre-iPSCs to iPSCs conversion. Mechanistically, lincRNA-p21 has been suggested to sustain the heterochromatic state at pluripotency gene promoters by interacting with HNRNPK. HNRNPK and lincRNA-p21 together would form a repressive complex able to preserve H3K9me3 and CpG methylation at the promoter of key pluripotency regulators such as Nanog, Sox2, and Lin28 [62] (Figure 3). Besides lincRNA-p21, there are only limited examples of nuclear lncRNAs regulating gene expression by controlling DNA methylation. In a more recent paper, Wang and colleagues reported the identification of Dum, a Developmental pluripotency-associated 2 (Dppa2) Upstream binding Muscle lncRNA [65]. LncRNA Dum was found to silence its neighboring gene Dppa2 in* cis* by recruiting Dnmt1, Dnmt3a and Dnmt3b on its promoter. Although the cited work was mainly focused on myogenic differentiation, it is tempting to speculate that a similar regulatory mechanism might play a role in pluripotent cells as well. Dppa2 is highly enriched in pluripotent cells and activation of endogenous Dppa2 during late steps of reprogramming specifically marks the small subset of cells that will achieve full pluripotency, in which Dppa2-mediated induction of Nanog transcription is a crucial event [66]. Therefore, it will be extremely interesting in the future to assess whether the lncRNA Dum regulates critical steps of reprogramming through modulation of Dppa2.

## 4. Concluding Remarks

Pluripotency is a unique property of ESCs and iPSCs, which are the only cell types able to undergo indefinite self-renewal and differentiation into derivatives of the three germ layers. Pluripotent cells therefore represent both ideal candidates for dissecting the mechanisms of early embryonic development and potential therapeutic tools for regenerative medicine. Patient-specific iPSCs also provide* in vitro* platforms to model human disease and to test drugs in preclinical studies. Such potential applications, however, are subordinated to a deep understanding of the molecular mechanisms underlying pluripotency.

An orchestra of transcription factors, chromatin regulators, signaling transducers, miRNAs and lncRNAs play coordinately in pluripotent cells. Each of them cannot be considered a solo player. Complex networks and feedback loops exist, which comprise members of each class of regulatory factors. A huge increase of transcriptome-wide analyses, facilitated by recent advancements in next-generation sequencing technologies, uncovered a universe of long noncoding transcripts. While there is no general consensus on the extent of the global impact of lncRNAs on the regulation of cell identity and differentiation, few examples in which selected lncRNAs have been more deeply analyzed exist. As discussed in this review, at least a subset of known lncRNAs are as important as previously defined “core transcription factors” in the context of pluripotent cells (Table 1). The paucity of functional studies is in striking contrast with the number of annotated lncRNAs (thousands) that are specifically enriched in ESCs and/or described as interactors of crucial pluripotency regulators, such as Polycomb and Trithorax complexes. We expect, in the near future, a substantial increase of functional studies describing new examples of lncRNAs acting in network with other master regulators in the definition of the pluripotent state.

## Figures and Tables

**Table 1:** lncRNA transcripts expressed in ESCs and controlling pluripotency.

lncRNA	Species	Proposed role in ESCs pluripotency	References
*AK028326 (GOMAFU/MIAT) *	m	Oct4-activated lncRNA which controls Oct-4 expression by a feedback loop.	[38]
*AK141205 *	m	Nanog-repressed lncRNA; AK141205 positively regulates Oct4 expression.	[38]
*ES1, ES2, ES3 *	h	Oct4 and Nanog transcriptional targets. ES1-3 act as modular scaffold for PRC2 and SOX2.	[40]
*MEG3/GTL2 *	m, h	Facilitates PRC2/JARID2 complex recruitment on target genes.	[47]
*lincRNA-1592, lincRNA-1552 *	m	Bind WDR5/MLL1 complex and are necessary for Nanog and Oct4 expression.	[52]
*MIRA *	m	Facilitates WDR5/MLL1 complex recruitment on chromatin.	[55]
*TUNA/MEGAMIND *	m	Activates transcription of Nanog, Sox2, and Fgf4.	[42]
*pRNA *	m	Recruits TIP5 on rDNA upon differentiation.	[56]
*linc-RoR *	h	Acts as a sponge for miR-145.	[60]
*lincRNA-p21 *	h	Interacts with HNRNPK to form a repressive complex at the promoter of key pluripotency regulators.	[62]
*Dum *	m	Favours DNA methylation at CpG sites at the promoter of the pluripotency-associated Dppa2.	[65]

Species: m = mouse; h = human.

## References

[1] Rinn J. L., Chang H. Y. (2012). Genome regulation by long noncoding RNAs. *Annual Review of Biochemistry*.

[2] Ulitsky I., Bartel D. P. (2013). lincRNAs: genomics, evolution, and mechanisms. *Cell*.

[3] Djebali S., Davis C. A., Merkel A. (2012). Landscape of transcription in human cells. *Nature*.

[4] Cabili M. N., Trapnell C., Goff L. (2011). Integrative annotation of human large intergenic noncoding RNAs reveals global properties and specific subclasses. *Genes and Development*.

[5] Ulitsky I., Shkumatava A., Jan C. H., Sive H., Bartel D. P. (2011). Conserved function of lincRNAs in vertebrate embryonic development despite rapid sequence evolution. *Cell*.

[6] Batista P. J., Chang H. Y. (2013). Long noncoding RNAs: cellular address codes in development and disease. *Cell*.

[7] Johnsson P., Lipovich L., Grandér D., Morris K. V. (2014). Evolutionary conservation of long non-coding RNAs; sequence, structure, function. *Biochimica et Biophysica Acta: General Subjects*.

[8] Diederichs S. (2014). The four dimensions of noncoding RNA conservation. *Trends in Genetics*.

[9] Derrien T., Johnson R., Bussotti G. (2012). The GENCODE v7 catalog of human long noncoding RNAs: analysis of their gene structure, evolution, and expression. *Genome Research*.

[10] Fatica A., Bozzoni I. (2014). Long non-coding RNAs: new players in cell differentiation and development. *Nature Reviews Genetics*.

[11] Tsai M.-C., Manor O., Wan Y. (2010). Long noncoding RNA as modular scaffold of histone modification complexes. *Science*.

[12] Morlando M., Ballarino M., Fatica A., Bozzoni I. (2014). The role of long noncoding RNAs in the epigenetic control of gene expression. *ChemMedChem*.

[13] Rinn J. L., Kertesz M., Wang J. K. (2007). Functional demarcation of active and silent chromatin domains in human HOX loci by noncoding RNAs. *Cell*.

[14] Wang K. C., Yang Y. W., Liu B. (2011). A long noncoding RNA maintains active chromatin to coordinate homeotic gene expression. *Nature*.

[15] Kino T., Hurt D. E., Ichijo T., Nader N., Chrousos G. P. (2010). Noncoding RNA Gas5 is a growth arrest- and starvation-associated repressor of the glucocorticoid receptor. *Science Signaling*.

[16] Tripathi V., Ellis J. D., Shen Z. (2010). The nuclear-retained noncoding RNA MALAT1 regulates alternative splicing by modulating SR splicing factor phosphorylation. *Molecular Cell*.

[17] Hung T., Wang Y., Lin M. F. (2011). Extensive and coordinated transcription of noncoding RNAs within cell-cycle promoters. *Nature Genetics*.

[18] Lai F., Orom U. A., Cesaroni M. (2013). Activating RNAs associate with Mediator to enhance chromatin architecture and transcription. *Nature*.

[19] Ounzain S., Pezzuto I., Micheletti R. (2014). Functional importance of cardiac enhancer-associated noncoding RNAs in heart development and disease. *Journal of Molecular and Cellular Cardiology*.

[20] Gong C., Maquat L. E. (2011). LncRNAs transactivate STAU1-mediated mRNA decay by duplexing with 39'UTRs via Alu elements. *Nature*.

[21] Faghihi M. A., Modarresi F., Khalil A. M. (2008). Expression of a noncoding RNA is elevated in Alzheimer's disease and drives rapid feed-forward regulation of *β*-secretase. *Nature Medicine*.

[22] Tay Y., Rinn J., Pandolfi P. P. (2014). The multilayered complexity of ceRNA crosstalk and competition. *Nature*.

[23] Cesana M., Cacchiarelli D., Legnini I. (2011). A long noncoding RNA controls muscle differentiation by functioning as a competing endogenous RNA. *Cell*.

[24] Memczak S., Jens M., Elefsinioti A. (2013). Circular RNAs are a large class of animal RNAs with regulatory potency. *Nature*.

[25] Ivanov A., Memczak S., Wyler E. (2015). Analysis of intron sequences reveals hallmarks of circular RNA biogenesis in animals. *Cell Reports*.

[26] Boyer L. A., Tong I. L., Cole M. F. (2005). Core transcriptional regulatory circuitry in human embryonic stem cells. *Cell*.

[27] Spivakov M., Fisher A. G. (2007). Epigenetic signatures of stem-cell identity. *Nature Reviews Genetics*.

[28] Rosa A., Brivanlou A. H. (2013). Regulatory non-coding RNAs in pluripotent stem cells. *International Journal of Molecular Sciences*.

[29] Guttman M., Donaghey J., Carey B. W. (2011). LincRNAs act in the circuitry controlling pluripotency and differentiation. *Nature*.

[30] Sauvageau M., Goff L. A., Lodato S. (2013). Multiple knockout mouse models reveal lincRNAs are required for life and brain development. *Elife*.

[31] Rosa A., Brivanlou A. H. (2009). MicroRNAs in early vertebrate development. *Cell Cycle*.

[32] Rosa A., Spagnoli F. M., Brivanlou A. H. (2009). The miR-430/427/302 family controls mesendodermal fate specification via species-specific target selection. *Developmental Cell*.

[33] Melton C., Judson R. L., Blelloch R. (2010). Opposing microRNA families regulate self-renewal in mouse embryonic stem cells. *Nature*.

[34] Takahashi K., Yamanaka S. (2006). Induction of pluripotent stem cells from mouse embryonic and adult fibroblast cultures by defined factors. *Cell*.

[35] Dinger M. E., Amara P. P., Mercer T. R. (2008). Long noncoding RNAs in mouse embryonic stem cell pluripotency and differentiation. *Genome Research*.

[36] Guttman M., Amit I., Garber M. (2009). Chromatin signature reveals over a thousand highly conserved large non-coding RNAs in mammals. *Nature*.

[37] Guttman M., Garber M., Levin J. Z. (2010). Ab initio reconstruction of cell type-specific transcriptomes in mouse reveals the conserved multi-exonic structure of lincRNAs. *Nature Biotechnology*.

[38] Mohamed J. S., Gaughwin P. M., Lim B., Robson P., Lipovich L. (2010). Conserved long noncoding RNAs transcriptionally regulated by Oct4 and Nanog modulate pluripotency in mouse embryonic stem cells. *RNA*.

[39] Khalil A. M., Guttman M., Huarte M. (2009). Many human large intergenic noncoding RNAs associate with chromatin-modifying complexes and affect gene expression. *Proceedings of the National Academy of Sciences of the United States of America*.

[40] Ng S.-Y., Johnson R., Stanton L. W. (2012). Human long non-coding RNAs promote pluripotency and neuronal differentiation by association with chromatin modifiers and transcription factors. *The EMBO Journal*.

[41] Loewer S., Cabili M. N., Guttman M. (2010). Large intergenic non-coding RNA-RoR modulates reprogramming of human induced pluripotent stem cells. *Nature Genetics*.

[42] Lin N., Chang K.-Y., Li Z. (2014). An evolutionarily conserved long noncoding RNA TUNA controls pluripotency and neural lineage commitment. *Molecular Cell*.

[43] Zhao J., Ohsumi T. K., Kung J. T. (2010). Genome-wide identification of polycomb-associated RNAs by RIP-seq. *Molecular Cell*.

[44] Davidovich C., Zheng L., Goodrich K. J., Cech T. R. (2013). Promiscuous RNA binding by Polycomb repressive complex 2. *Nature Structural and Molecular Biology*.

[45] Peng J. C., Valouev A., Swigut T. (2009). Jarid2/Jumonji coordinates control of PRC2 enzymatic activity and target gene occupancy in pluripotent cells. *Cell*.

[46] Shen X., Kim W., Fujiwara Y. (2009). Jumonji modulates polycomb activity and self-renewal versus differentiation of stem cells. *Cell*.

[47] Kaneko S., Bonasio R., Saldaña-Meyer R. (2014). Interactions between JARID2 and noncoding RNAs regulate PRC2 recruitment to chromatin. *Molecular Cell*.

[48] Takahashi N., Okamoto A., Kobayashi R. (2009). Deletion of Gtl2, imprinted non-coding RNA, with its differentially methylated region induces lethal parent-origin-dependent defects in mice. *Human Molecular Genetics*.

[49] Zhou Y., Cheunsuchon P., Nakayama Y. (2010). Activation of paternally expressed genes and perinatal death caused by deletion of the Gtl2 gene. *Development*.

[50] Stadtfeld M., Apostolou E., Akutsu H. (2010). Aberrant silencing of imprinted genes on chromosome 12qF1 in mouse induced pluripotent stem cells. *Nature*.

[51] Ang Y.-S., Tsai S.-Y., Lee D.-F. (2011). Wdr5 mediates self-renewal and reprogramming via the embryonic stem cell core transcriptional network. *Cell*.

[52] Yang Y. W., Flynn R. A., Chen Y. (2014). Essential role of lncRNA binding for WDR5 maintenance of active chromatin and embryonic stem cell pluripotency. *eLife*.

[53] Kessel M., Balling R., Gruss P. (1990). Variations of cervical vertebrae after expression of a Hox-1.1 transgene in mice. *Cell*.

[54] Kostic D., Capecchi M. R. (1994). Targeted disruptions of the murine Hoxa-4 and Hoxa-6 genes result in homeotic transformations of components of the vertebral column. *Mechanisms of Development*.

[55] Bertani S., Sauer S., Bolotin E., Sauer F. (2011). The noncoding RNA Mistral activates Hoxa6 and Hoxa7 expression and stem cell differentiation by recruiting MLL1 to chromatin. *Molecular Cell*.

[56] Savić N., Bär D., Leone S. (2014). LncRNA maturation to initiate heterochromatin formation in the nucleolus is required for exit from pluripotency in ESCs. *Cell Stem Cell*.

[57] Li M., Liu G.-H., Belmonte J. C. I. (2012). Navigating the epigenetic landscape of pluripotent stem cells. *Nature Reviews Molecular Cell Biology*.

[58] Menendez S., Camus S., Belmonte J. C. I. (2010). p53: guardian of reprogramming. *Cell Cycle*.

[59] Zhang A., Zhou N., Huang J. (2013). The human long non-coding RNA-RoR is a p53 repressor in response to DNA damage. *Cell Research*.

[60] Wang Y., Xu Z., Jiang J. (2013). Endogenous miRNA sponge lincRNA-RoR regulates Oct4, nanog, and Sox2 in human embryonic stem cell self-renewal. *Developmental Cell*.

[61] Xu N., Papagiannakopoulos T., Pan G., Thomson J. A., Kosik K. S. (2009). microRNA-145 regulates OCT4, SOX2, and KLF4 and represses pluripotency in human embryonic stem cells. *Cell*.

[62] Bao X., Wu H., Zhu X. (2015). The p53-induced lincRNA-p21 derails somatic cell reprogramming by sustaining H3K9me3 and CpG methylation at pluripotency gene promoters. *Cell Research*.

[63] Huarte M., Guttman M., Feldser D. (2010). A large intergenic noncoding RNA induced by p53 mediates global gene repression in the p53 response. *Cell*.

[64] Stadtfeld M., Maherali N., Breault D. T., Hochedlinger K. (2008). Defining molecular cornerstones during fibroblast to iPS cell reprogramming in mouse. *Cell Stem Cell*.

[65] Wang L., Zhao Y., Bao X. (2015). LncRNA Dum interacts with Dnmts to regulate Dppa2 expression during myogenic differentiation and muscle regeneration. *Cell Research*.

[66] Buganim Y., Faddah D. A., Cheng A. W. (2012). Single-cell expression analyses during cellular reprogramming reveal an early stochastic and a late hierarchic phase. *Cell*.

